# Provider and lay perspectives on intra-uterine contraception: a global review

**DOI:** 10.1186/s12978-017-0380-8

**Published:** 2017-09-26

**Authors:** Marina A. S. Daniele, John Cleland, Lenka Benova, Moazzam Ali

**Affiliations:** 1 Faculty of Epidemiology and Population Health, London School of Hygiene & Tropical Medicine, Keppel Street, London, WC1E 7HT UK; 2 Department of Reproductive Health and Research, World Health Organization, Geneva, Switzerland

**Keywords:** Intra-uterine contraception (IUC), Intra-uterine device (IUD), Health-care providers, Users, Lay perspectives

## Abstract

**Background:**

Intra-uterine contraception (IUC) involves the use of an intra-uterine device (IUD), a highly effective, long-acting, reversible contraceptive method. Historically, the popularity of IUC has waxed and waned across different world regions, due to policy choices and shifts in public opinion. However, despite its advantages and cost-effectiveness for programmes, IUC’s contribution to contraceptive prevalence is currently negligible in many countries.

This paper presents the results of a systematic review of the global literature on provider and lay perspectives on IUC. It aims to shed light on the reasons for low use of IUC and reflect on potential opportunities for the method’s promotion.

**Methods:**

A systematic search of the literature was conducted in four peer-reviewed journals and four electronic databases (MEDLINE, EMBASE, POPLINE, and Global Health). Screening resulted in the inclusion of 68 relevant publications.

**Results:**

Most included studies were conducted in areas where IUD use is moderate or low. Findings are similar across these areas. Many providers have low or uneven levels of knowledge on IUC and limited training. Many wrongly believe that IUC entails serious side effects such as pelvic inflammatory disease (PID), and are reluctant to provide it to entire eligible categories, such as HIV-positive women. There is particular resistance to providing IUC to teenagers and nulliparae. Provider opinions may be more favourable towards the hormonal IUD. Some health-care providers choose IUC for themselves.

Many members of the public have low knowledge and unfounded misconceptions about IUC, such as the fear of infertility. Some are concerned about the insertion and removal processes, and about its effect on menses. However, users of IUC are generally satisfied and report a number of benefits. Peers and providers exert a strong influence on women’s attitudes.

**Conclusion:**

Both providers and lay people have inaccurate knowledge and misconceptions about IUC, which contribute to explaining its low use. However, many reported concerns and fears could be alleviated through correct information. Concerted efforts to train providers, combined with demand creation initiatives, could therefore boost the method’s popularity. Further research is needed on provider and lay perspectives on IUDs in low- and middle-income countries.

**Electronic supplementary material:**

The online version of this article (10.1186/s12978-017-0380-8) contains supplementary material, which is available to authorized users.

## Plain English summary

This article focuses on intra-uterine contraception (IUC), which involves the use of an intra-uterine device (IUD), a highly effective contraceptive method. The proportion of contraceptive users choosing the IUD varies across the world, but overall it is little used. In this article, we aimed to summarise what is known about the opinions of health-care providers and members of the public concerning IUC. Our aim was to better understand why its use is low and whether there is any scope to promote it further. We conducted a rigorous search of scientific journals and electronic databases in order to identify relevant articles, and found 68.

These studies suggest that many health-care providers are reluctant to recommend the method because they wrongly believe it has negative side effects. Although teenagers and women with no children can use IUC safely, many providers are unwilling to provide it to them. Many members of the general public have low knowledge or misunderstand how IUC works, for example they fear that IUDs cause infertility. Others are worried about how the device is inserted, or about how it alters the menstrual cycle. However, women using IUC are generally satisfied.

These findings are remarkably similar in studies from across the world, and partly explain why the use of IUDs is low. However, they also suggest that providing correct information could reassure people about the method's safety. Therefore, better training for health-care providers and awareness raising among the general public could increase IUC’s popularity.

## Background

Intra-uterine contraception (IUC) involves the use of an intra-uterine device (IUD), a form of long-acting, reversible contraception (LARC). IUDs are among the most effective contraceptive methods, with 8 pregnancies per 1000 women in the first year of use for the copper-bearing IUD, and 2 per 1000 women for the levonorgestrel-releasing IUD or LNG-IUD (also known as the intra-uterine system or IUS) [1]. The copper-bearing IUD is effective for 10–12 years, and the LNG-IUD for 5 years, both with immediate fertility return once removed [2]. Because they need to be inserted and removed by a health-care provider, the likelihood of user error is limited. These features make IUDs highly cost-effective for programmes [3, 4]. IUDs are suitable for groups who may have difficulty accessing contraception, such as adolescents [5] and HIV-positive women [6, 7]. The copper-bearing IUD is also useful for women wishing to avoid side-effects associated with hormonal contraception, whereas the LNG-IUD, producing lighter periods, is protective against anaemia [2]. Furthermore, IUC can be used postpartum [8] and postabortion [9], and is also the most effective form of emergency contraception [10].

Today, although IUC is used by large numbers of women, its global distribution is uneven [11]. In a few countries, including China and most of Central Asia, IUDs constitute at least half of all contraceptive use. In Northern Africa and the Middle East, they represent about a quarter of all use, and in parts of Europe, about a fifth. In the past two decades, levels have remained broadly constant or declined slightly in these areas. Conversely, in the Americas, IUC’s share of use is generally well below 10%, despite a spike in the USA in recent years. Finally, in sub-Saharan Africa, South Asia and the Pacific region, the contribution of IUDs to the method mix is minimal [11]. In sub-Saharan Africa, although increasing numbers of women wish to limit future births [12], the majority are using short-acting methods [13], and the current rise in contraceptive prevalence is largely driven by injectables [14].

Two interacting factors are most helpful in explaining this degree of geographical variation. Firstly, early programmatic choices by certain governments dramatically shaped the contraceptive mix available to whole generations. For example, in the Soviet Union, the IUD was promoted because it was locally produced and affordable [15], while elsewhere the lack of foreign aid, or the preferences of donors, influenced the method mix [16].

Secondly, contraceptive methods fall in and out of favour among health providers and clients. Reverberations of the 1970s Dalkon Shield scandal were felt in the US for several decades, and led to the virtual disappearance of IUDs [17]. The dominance of certain methods limits local providers’ knowledge and familiarity with others, leading to a reluctance to recommend them. In the case of IUDs, this is particularly relevant because health-care providers need to undergo special training and to practice insertion regularly in order to maintain skills and confidence. In turn, the opinion of providers, coupled with the exposure to experienced peers, has a strong impact on potential users’ views [18].

It has long been acknowledged that the availability of a range of contraceptive methods is most likely to meet the needs of individuals [19]. People may be reluctant to start using a method unless they know of and can access one that meets their requirements. In addition, if only a small number of methods are available, those experiencing side-effects may abandon contraception altogether [20]. Increasing the number of contraceptive options, therefore, tends to drive up overall use [21]. IUDs, with their specific features, are a valuable component of the method mix. Exploring provider and lay/user preferences regarding IUC can shed light on current patterns of use, and illustrate the potential which exists for its promotion in countries where it is little used.

Existing reviews on IUC have covered barriers to uptake more generally, with an emphasis on access [22], or have focused on provision to adolescents [23]. This article is based on a systematic search of the literature which was conducted as part of a broader research project on IUC [24]. Drawing on a relevant subset of results, it aims to comprehensively summarise and interpret recent global findings on health provider and lay perspectives on use of IUC.

## Methods

A search was conducted in mid-December 2015 with the aim to retrieve articles from peer-reviewed journals and grey literature, published from 2010 to 2015 in any country, concerning provider and lay perspectives on IUC, facilitators and barriers, or describing interventions aimed at increasing the uptake and continued use of the method. “Provider perspectives” are those of all cadres of person providing health-care services, including in facilities and in the community. “Lay perspectives” are those of all people who are not health-care providers, including but not limited to users and potential users of contraception. We searched Medline, Popline, Embase and Global Health electronic databases using keywords and subject headings. This search was preceded by a manual search of four journals (Contraception, the European Journal of Family Planning and Reproductive Health Care, Perspectives on Sexual and Reproductive Health, and International Perspectives on Sexual and Reproductive Health) in order to establish key words that would capture the relevant literature. For the database searches, no limitation was set on language, but search terms were in English. Where possible, a limitation was set on keywords being present in the Title or Abstract, and to human subjects. For the detailed search strategy, see Additional file 1.

**Fig. 1:**
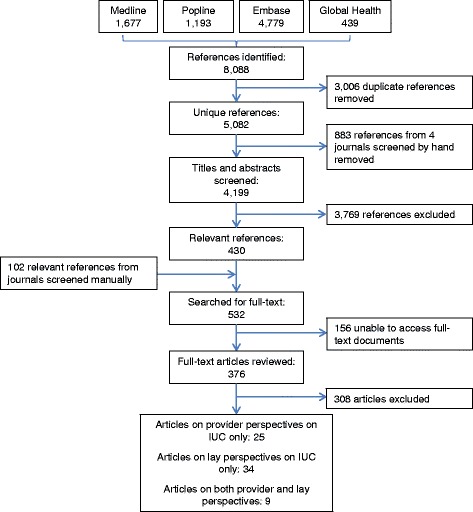
Search flowchart

Following the elimination of duplicates, the titles and abstracts of 4199 records were individually screened by MD and LB. Studies of safety, efficacy, contraindications and clinical management, and of IUC as emergency contraception were excluded. Studies were retained based on their thematic relevance only, and the quality of the evidence was not systematically graded. The searches resulted in a total of 532 relevant references (Fig. 1). Excluded articles did not contain specific information related to use of IUC or IUC as part of LARC, or were clinical or biomedical in nature. From the 376 publications for which full texts were reviewed, 68 original studies were included in this review. These were all cross-sectional, descriptive surveys, or qualitative studies. Nine studies reported perspectives of both providers and lay people, 25 studies reported provider perspectives only and 34 studies reported lay people’s perspectives only. Details of the studies are in Additional file 2.

## Results

### Provider perspectives

A total of 34 studies on provider perspectives were identified (including 9 which also reported on lay perspectives). These included 11 publications from the USA and 13 from Asia, Latin America and Africa. A further 7 studies related to European and other high-income countries. Three papers were based on multi-country data.

### Knowledge, training and attitudes

Our findings show that many providers across the world have low or uneven levels of knowledge, limited training, and negative attitudes towards IUC, although some choose the method for themselves.

Qualitative studies report that in Cambodia, village health workers share very much the same misconceptions about IUDs as the villagers themselves [25], whereas in Pakistan, insufficient training is the main reason for providers’ reluctance to provide IUDs, alongside perceived lack of demand [26]. Complaints about lack of training were also a common theme in two studies conducted in Ghana [27, 28]. For example, most physicians and midwives at a Kumasi teaching hospital reported limited knowledge, and had never inserted an IUD [27]. In South Africa, 32 providers at public sector clinics reported a need for further training, and very few spontaneously mentioned IUC as a method that they would recommend [29]. Where specific training has been provided, however, it appears to have a positive impact on attitudes. A study of 27 providers from Kenya, who had been specially trained by the local affiliate of Marie Stopes International (MSI) in the provision of LARC, documented positive attitudes towards the LNG-IUD [30].

Providers’ own use of IUC is high in certain settings. For example, 61% of German obstetrician/gynaecologists (OB/GYNs) would choose the LNG-IUD for themselves [31]. LNG-IUD was the most used method among 1001 surveyed OB/GYNs and general practitioners (GPs) from ten middle-high income countries [32]. An association was found between the methods providers used and those they would recommend to clients. However, use of IUC by professionals may not directly correlate with the method’s broader popularity. A nationally representative survey in Spain found that use of IUC among health-care providers was almost double the rate seen among the general population [33].

### Concerns about side effects

One reason why providers are reluctant to recommend IUC may be the belief that the method is linked to serious side effects.

For example, about half of 385 surveyed private sector providers in Bangladesh were of the opinion that IUDs had too many, or too serious, side effects to recommend [34]. A common concern is the belief in a link between IUDs and pelvic inflammatory disease (PID), reported in papers from the US [35–37] and El Salvador [38]. Misconceptions about a link to both PID and to infertility were prevalent among providers in Pakistan [26]. A nationally representative survey of 765 French OB/GYNs and GPs found that a majority believe IUC to be a risk factor for PID, and also for ectopic pregnancy [39]. Finally, a small South African enquiry noted that two-thirds of providers thought that the copper-bearing IUDs represented serious health risks including PID, ectopic pregnancy, and uterine perforation [29].

### Provision to young and nulliparous women

The WHO’s medical eligibility criteria for contraceptive use recommend copper-bearing IUD and LNG-IUD use by nulliparous and young women [40]. However, there is abundant evidence suggesting that providers are particularly reluctant to provide IUC to these groups.

Low willingness to provide IUC to young/nulliparous women has been documented in several US-based surveys of practitioners [35, 41–45]. For example, according to a nationally representative survey, only 30% of family doctors would consider IUC for a teenager and 43% for a nulliparous patient [41]. The corresponding estimates among OB/GYNs were 53% and 71%, indicating greater awareness of eligibility criteria among this specialist group. Similar differences in awareness between professional categories were reported in several other US studies [35, 42–44]. One exception is a survey reporting that only 30% of physicians and nurses have misconceptions about the safety of IUC for nulliparous women [45].

The unwillingness to provide IUC to young/nulliparous women is not limited to the US. In Nepal, a survey of 345 nurses and midwives established that knowledge of IUC was uneven with regard to eligibility criteria [46]. A large majority (83%) of French doctors consider IUC to be contraindicated for nulliparous women [39], and Norwegian GPs rarely counsel young women on the method [47]. Nationally representative surveys of 1444 clinicians (mostly nurses) showed that in South Africa, 25% would consider IUDs for adolescents and 33% for nulliparous women, whereas in Zimbabwe the corresponding estimates were 10% and 12% [48]. Providers in sub-Saharan African cities may be somewhat less reluctant: about half of surveyed clinicians working at a teaching hospital in Ghana would recommend an IUD for a teenager or for a nulliparous woman [27]. Similarly, among 676 urban providers (mostly nurses) in Kenya, between 11% and 20% restrict IUD access on the grounds of parity or marital status, or require prior consent from a third party [49].

A gradient has been observed in the willingness of practitioners to recommend IUC, increasing by age and parity. A small study in the US neatly reported that whereas only 27% of GPs would recommend IUC for a nulliparous teenager, this figure rose to 50%, 60% and 77% for a nulliparous 21 year-old, a postpartum teenager, and a breastfeeding 21 year old, respectively [50]. In Sweden, willingness to consider IUC as an option rose linearly with patients’ age [51].

Attempts have been made to explore why providers are so concerned about providing IUC to these groups. An online survey of 1862 providers from 15 countries, mainly OB/GYNs, showed that the main barriers to providing IUC to nulliparous women were perceived difficulty of insertion, concerns about pain, PID, and infertility [52]. Almost identical concerns were apparent in a survey of 1103 contraceptive providers in seven, mostly European, countries [53]. In addition, one of the few qualitative studies on this topic showed that a concern about high discontinuation of LARC methods among young women, as well as about the length of counselling needed, may act as disincentives among US professionals [54].

As far as the LNG-IUD is concerned, a multi-country study in middle and high-income countries showed that most providers would recommend an LNG-IUD for limiting, but not for spacing [32]. Similarly, 42% of 2016 German OB/GYNs would never consider an LNG-IUD for a nulliparous woman [31]. However, there is also evidence that providers may be more willing to provide the LNG-IUD to nulliparae, compared to the copper-bearing IUD. Among OB/GYN attendees at an international conference in Chile, while 80% would not make copper-bearing IUDs available to nulliparous women, only 10% would not provide an LNG-IUD [55]. Similarly, a survey of 701 Australian OB/GYNs revealed that whereas only 39% believed copper-bearing IUDs to be suitable for nulliparae, this figure rose to 69% for LNG-IUDs [56].

### Provision to other categories of women

The literature also shows that some providers are reluctant to provide IUC to other categories of women who may benefit from the method.

In South Africa and Zimbabwe, for example, 95% of providers consider IUDs inappropriate for those at risk of HIV or HIV-positive [48]. Reluctance is also reported in relation to postpartum provision. US studies indicated very low awareness of immediate postpartum or postabortion insertion of IUC as a sound strategy [42–44], and only 36% of providers at a teaching hospital in Ghana said they would recommend immediate postpartum insertion [27].

Finally, a US study showed that the propensity of providers to recommend IUC was conditioned by the ethnicity and social status of patients [57]. Health-care providers were more likely to recommend IUC to black and Latina women than to white women, although, paradoxically, among whites they offered it more frequently to higher status patients.

### User/lay perspectives

A total of 43 studies on lay perspectives were identified (including 9 which also reported on provider perspectives). This literature is dominated by research from the USA (18 publications), followed by studies from Africa, Latin America and Asia (14), and from European and other high-income countries (11).

### Low knowledge and misconceptions

The findings of this review suggest that many women and their partners have low levels of knowledge of IUC and have unfounded misconceptions about the method.

Several US studies found poor knowledge. Surveys have found that 55% of young family planning clients have not heard of the IUD [58], and only 20% of women attending primary care know that IUC is more effective than oral contraception [59]. Two papers used survey data on young men, finding very poor knowledge on IUC [60, 61]. In one of these, nearly half of participants believed that IUDs were banned in the US [61].

Qualitative studies in low/middle-income countries showed a range of concerns about health risks associated with IUDs, including cancer, ectopic pregnancy, infertility and harm to the husband during sex [25, 26, 62–65]. According to a large survey of women and men in Pakistan, the main concern was that IUDs will harm the womb [66], and two large surveys in Ethiopia ascertained that one of the main reasons for not using LARC methods was the fear of infertility [67, 68]. Concerns with fertility impairment were also voiced by the majority of respondents to a national survey of young women in France [69], and in a mixed-methods study in the US [70]. A UK survey of 502 young women documented more negative attitudes towards IUC than towards injectables or implants, although all three methods invoked concerns about fertility impairment [71].

While discreetness is considered to be a positive feature by French users of IUC [72], studies from around the world report that many other women are concerned about the fact that the IUD is positioned inside the body. The authors of a study conducted on the Thai-Burmese border concluded that this idea fostered imagined and exaggerated fears [63]. A US-based online survey of non-IUD users found that the position of the device worried them [73], a concern also emerging from several qualitative studies from the US [54, 74–76] and Europe [77]. A small study of abortion patients in Scotland found that 24% believed that the device could move around the body [78].

### Concerns about insertion and removal

Findings suggest that some women are concerned about procedures for insertion and removal of IUC and about the need for involvement of a health-care provider.

Among 617 postpartum women surveyed in Kenya, the major reasons for choosing an implant over an LNG-IUD were less pain, less infringement of modesty, and preference for a superficial insertion in the arm [79]. Pain during insertion is also a concern identified in qualitative and mixed-methods studies from the US and Europe [54, 70, 71, 74–77]. In Scotland, 34% of abortion patients in a small survey thought the insertion would be painful [78].

A US-based online survey of non-IUD users found that the need for provider insertion and removal is itself considered a disadvantage [73], and two surveys of abortion-seekers in the US found that self-removal of IUC would enhance the appeal of the method [80, 81]. In Cambodia, women were concerned that staff would refuse to remove the device [25].

### Benefits and disadvantages of IUC compared to other methods

Compared to studies seeking the views of the general population, those focused on women currently using or interested in using IUC report that the method has several perceived advantages and few disadvantages.

Qualitative studies from Cambodia, Pakistan, Nigeria, Madagascar and the Thailand-Burma border showed that users appreciated IUDs because of their effectiveness and long-acting nature [25, 26, 62–65]. These qualities were among the main positive features mentioned by users in six surveys conducted in the US, the UK, Australia and Pakistan [73, 82–84]. The method’s forgettability also contributed to satisfaction among users of IUC in the US and France [72, 85].

In addition, a qualitative study of users and non-users in seven European countries found that the perceived advantages of the LNG-IUD include its compatibility with breastfeeding and a quick return to fertility upon removal [77]. Rapid return to fertility was also mentioned in the study from Nigeria [62], while in Kenya, postpartum women choosing LNG-IUD over implants believed that the method had fewer side effects [79]. On the other hand, respondents from the UK and other European countries reported the lack of protection against STIs as a disadvantage of IUC [77, 84].

Finally, affordability was mentioned among the positive features of IUDs by users in Pakistan [86, 87], whereas the high cost of the LNG-IUD constituted a barrier for European women [77].

### The effect on menses

The literature suggests that women’s preferences may vary in relation to the effect of the different types of IUDs on menses.

One of the most comprehensive US surveys suggests that women who want to avoid irregular bleeding are less likely to choose the copper-bearing IUD [82]. Menstrual irregularity was the main complaint among users of IUC in a survey in France, though the type of device was not specified [72], and acted as a disincentive preventing young women in the UK from choosing LARC methods [71]. A qualitative study found that some US women choosing an LNG-IUD welcomed the possibility of amenorrhea, while those choosing a copper-bearing IUD were motivated by a desire to retain regular menses [85]. While light, less painful periods are among the most desirable features of contraceptive methods according to Australian women [83], the suppression of regular menses by the LNG-IUD evokes mixed feelings among teens and Latina women in the US [54, 88], and is seen as a disadvantage in Europe [77]. However, a cohort study of postpartum women in Kenya found that LNG-IUD users were more likely to find their bleeding pattern highly acceptable, compared to implant users [89].

### The influence of peers and providers

Our findings suggest that peers and providers strongly influence women’s decision to use IUC.

In the US, a survey of abortion clients showed that disclosure of personal use of IUC by a provider increased their likelihood of choosing this method [90]. Another US-based survey of women of reproductive age found that the few women who had been counselled on IUC and those who had talked to a user of IUC were more knowledgeable about the method [59].

However, qualitative studies from low-income settings have shown that while providers are regarded as useful sources of information, reassurance from friends or relatives who have used IUDs is a crucial factor in attitude formation [62–64]. In Madagascar, the availability of a family planning counsellor or friend to accompany the woman to the clinic for insertion was also important [64]. US-based qualitative studies show that social networks are a valued source of information, though negative conversations may be more memorable than positive ones [76, 91].

On the other hand, a survey conducted in five European countries found that male partners have relatively little influence on choice of IUC, given that about 80% of women do not involve their partners in decisions to use female-controlled methods [92]. Finally, a survey of 261 parents or guardians from the US found that IUC was the least acceptable method, among seven, for their teenage daughter [93].

## Discussion

This study aimed to summarise recent research findings on provider and lay perspectives regarding IUC. Results show that while some health-care providers have up-to-date knowledge on IUC and most users are satisfied, a negative reputation of the method persists. Many health-care providers lack training or regular opportunities for inserting IUDs and are therefore reluctant to provide them, and many women have misconceptions or are concerned about side effects. Most included studies were conducted in areas where IUD use is moderate or low. The biases and prejudices that providers and lay people have against the IUD are remarkably similar across countries regardless of income level, and contribute to explaining low uptake. They also shed light on the low levels of use of IUC compared with implant uptake in projects that promote both methods equally [94, 95].

Provider-imposed restrictions of access to contraceptive methods have been abundantly described in the contraceptive literature [96]. Barriers described include marriage or minimum-age restrictions, arbitrary schedules for return visits, the requirement that women be menstruating, and a variety of process hurdles [97–99]. Some providers may still adhere to outdated national regulations, such as system-level restrictions on IUDs and female sterilisation based on marital status [100].

It is likely that provider reluctance to provide IUC is due to a combination of outdated knowledge and personal bias, stemming in part from the fact that IUD insertion is more complex and takes longer than providing other reversible methods. This may explain providers’ resistance to providing the method to young women, despite the recent statements by WHO and US professional organisations on the suitability of IUC for adolescents [40, 101, 102]. It is difficult for new evidence to change ingrained mentalities, as demonstrated also by the refusal to provide HIV-positive women with IUDs [103, 104] and by the persistent obsession with the IUD-PID link [105]. Until outdated conceptions of eligibility are dispelled, whole categories of potential users will be excluded [17].

However, there is probably a large untapped potential to improve provider attitudes towards IUDs by ensuring that all professionals involved in family planning receive regular technical updates and in-service training [106, 107]. In high-income countries, OB/GYNs are more likely than general practitioners and nurses to receive regular updates on contraceptive safety, which may explain the different levels of knowledge between these professional categories. The advent of the LNG-IUD has revived interest in IUC in the USA and Europe, probably explaining its high uptake among health professionals themselves and an apparent greater inclination to provide it. However, the price of LNG-IUD would need to be reduced for it to become a viable option for low-income countries [108].

Evidence from sub-Saharan Africa confirms the scarcity of trained and competent professionals in LARC method provision, particularly in rural areas [109–111]. It is important that national curricula accommodate sufficient time for practical and theoretical training [112], and this should be offered to all suitable cadres, including nurses and midwives [113]. However, the fact that most initiatives to promote LARC methods have so far been driven by NGOs rather than national governments has probably limited progress in this area [24].

All contraceptive methods, including IUC, have advantages and disadvantages. Women reach a rational decision on contraceptive uptake and method choice largely based on their perception of potential harm, alongside other considerations including economic, psychological, social, familial and personal costs [114, 115].

Among the general population, the existence of unfounded fears and rumours about modern contraceptive methods has been widely reported in the literature [97, 110, 111, 116]. However, our findings suggest that misconceptions concerning IUC are largely due to an inadequate understanding of the mechanism of action, suggesting that attitudes are likely to be amenable to improvement through sex education and contraceptive counselling. Providing accurate information can increase demand for contraception [117], and the uptake of more effective methods [11]. Regardless of the setting, informing women of the side-effects they might experience, and especially of the effect on menses, can increase satisfaction [118], which may, in turn, promote continuation [20].

However, the formation of attitudes towards contraception and the choice of IUC over other methods are also influenced by social networks. An analysis of DHS data from six African countries has shown that the likelihood of using contraception is strongly influenced by how a woman perceives that other female members of her community will judge her actions [119]. The provision of accurate information alone may therefore not be sufficient to improve attitudes, and exposure to the experiences of other people, even friends of friends, may be necessary [120]. Media campaigns and targeted awareness raising in communities may also be useful.

Although our findings show that similar opinions of IUC are reported by lay people across the world, the preference for certain methods may also vary by cultural or geographical context. For example, it has been suggested that injectables and implants will be preferred to IUC where modesty is an important concern, and that the LNG-IUD would not be popular where amenorrhea in young women is culturally unacceptable [112]. By contrast, women in Bangladesh have reported dissatisfaction with the copper-bearing IUD, because the increased menstrual flow causes disruption to their daily life [121].

In addition, we found some evidence that concerns about quality of care and mistrust of facilities may also limit uptake of IUC. Examples from the literature on low- and middle-income countries confirm that perceived high quality of care, and especially trust in health-care providers, result in higher contraceptive uptake and continuation [122, 123]. In order to attract users, improvements are therefore required across the various dimensions of quality of care, from the knowledge and counselling skills of providers, to the safety and hygienic standards of facilities [19].

Finally, the review has identified substantial gaps in the evidence. In low- and middle-income countries, research data on provider and lay perspectives on IUC and other methods is very patchy. In view of the scale of investment in family planning in these countries, it is astonishing how little attention has been paid to ascertaining the views of women on specific properties of contraceptive methods and what considerations are most important in choosing a method. Similarly, further studies on provider knowledge, beliefs, attitudes, and personal use regarding specific methods are justified. For example, papers from USA and Europe indicate that the method chosen by providers for their own use influences their recommendations to women. Yet almost nothing is known about the contraceptive choices made by many thousands of family planning staff in Asia, Africa, and Latin America.

## Conclusions

This review systematically explored recently published literature about provider and lay perspectives on IUC. It reveals overall low or inaccurate knowledge and misconceptions about the method as reported by studies from across the world, but good levels of satisfaction among users. Given that many people’s negative opinions of IUDs are based on incorrect information, it seems likely that concerted efforts to improve awareness and understanding of IUC among women and in communities would boost its popularity. At the same time, increased training and on-the-job support for providers would increase their willingness to recommend the method, thus stimulating demand. Further research is needed on provider and lay perspectives on IUDs and other methods, especially in low- and middle-income countries.

## Additional file

Additional file 1: Search strategy. (DOCX 12 kb)
Additional file 2: Detail of included studies. (DOCX 20 kb)

## Abbreviations

GP: General practitioner
IUC: Intra-uterine contraception
IUD: Intra-uterine device
LARC: Long-acting reversible contraception
OB/GYN: Obstetrician/gynaecologist
PID: Pelvic Inflammatory disease
