# Supplementary material for: Provider and lay perspectives on intra-uterine contraception: a global review
Source: Reprod Health. 2017 Sep 26;14:119. doi: 10.1186/s12978-017-0380-8 (PMC5615438; doi:10.1186/s12978-017-0380-8)
Supplement: Additional file 1: — Search strategy. (DOCX 12 kb) [file 12978_2017_380_MOESM1_ESM.docx]

**Additional File 1: Search strategy**

| Medline | IUD OR LNG-IUD OR LNG-LNG-IUD OR "Copper-bearing T" OR "Multiload Cu375" OR "Mirena" OR "intrauterine device" OR "intrauterine system" OR "intrauterine contraception" OR "Levonorgestrel-releasing intrauterine system"  Limits: Human, 2010-Current, term in Title or Abstract | Intrauterine Devices/ OR  Intrauterine Devices, Copper-bearing/ OR  Intrauterine Devices, medicated  Limits: Human, 2010-Current | **1677 results** |
| --- | --- | --- | --- |
| Popline | IUD (topic under Contraceptive methods) | No | **1193 results** |
| Embase | IUD OR LNG-IUD OR LNG-LNG-IUD OR "Copper-bearing T" OR "Multiload Cu375" OR "Mirena" OR "intrauterine device" OR "intrauterine system" OR "intrauterine contraception" OR "Levonorgestrel-releasing intrauterine system"  Limits: Human, 2010-Current, term in Title or Abstract | Intrauterine contraceptive device/ OR  Copper-bearing intrauterine device/ OR  Levonorgestrel releasing intrauterine system  Limits: Human, 2010-Current | **4779 results** |
| Global Health | IUD OR LNG-IUD OR LNG-LNG-IUD OR "Copper-bearing T" OR "Multiload Cu375" OR "Mirena" OR "intrauterine device" OR "intrauterine system" OR "intrauterine contraception" OR "Levonorgestrel-releasing intrauterine system"  Limits: 2010-Current | Intrauterine devices  Limits: 2010-Current  **279 results** | **439 results** |
|  |  |  | **TOTAL:**  **8088 results** |
