# Supplementary material for: Provider and lay perspectives on intra-uterine contraception: a global review
Source: Reprod Health. 2017 Sep 26;14:119. doi: 10.1186/s12978-017-0380-8 (PMC5615438; doi:10.1186/s12978-017-0380-8)
Supplement: Additional file 2: — Detail of included studies. (DOCX 20 kb) [file 12978_2017_380_MOESM2_ESM.docx]

| **Author, year** | **Population** | **Setting** | **Study design** | **Sample size** |
| --- | --- | --- | --- | --- |
| Adjei, 2015 | Health-care providers | Ga East municipality, Ghana | Cross-sectional survey and in-depth interviews | 51 health facilities (randomly sampled, data collected from all), facility checklist and interview with manager |
| Agha, 2010 | Lay people | Pakistan | Representative household survey | 1788 non-pregnant wives and 1805 husbands with non-pregnant wives, from separate households (response rate not reported) |
| Amin, 2012 | Lay people | Peshawar, Pakistan | Survey | 104 women attending a family planning centre (response rate not reported) |
| Anderson et al, 2014 | Lay people | San Francisco, USA | Qualitative interviews and focus groups | Women attending FP clinics and from the community: 24 interviews and 3 focus groups with 14 participants |
| Azmat et al, 2013 | Lay people | Sindh and Punjab provinces, Pakistan | Cross-sectional survey | 681 women who had accessed MSI mobile outreach programme (sampled from client list, no refusals reported) |
| Babalola and John, 2012 | Health-care providers & lay people | Benue and Oyo States, Nigeria | Multimethod qualitative study (focus group discussions (FGDs), in-depth interviews, key informant interviews (IDIs), free-listing interviews and pile-sorting exercises | in each of 6 communities: 4 FGDs with men and women (202 people in total), 2 IDIs with service providers (12 in total). In total, interviews with 10 key informants, 169 people conducted pile-sorting and 224 free-listing. |
| Bahamondes et al, 2015 | Health-care providers | OB/GYNs from 12 Latin American countries | Survey | 210 OB/GYNs (=98% response rate) |
| Benson et al, 2012 | Lay people | San Francisco, USA | Survey | 299 women undergoing induced abortion (=95% completed at least one half of the questionnaire and 72% both halves) |
| Bharadwaj et al, 2012 | Lay people | London, UK | Survey | 194 women attending an integrated young people service (=95% response rate) |
| Binkowska and Harvey, 2010 | Lay people | France, Germany, Italy, Poland, Russia, Spain and Turkey | Focus group discussions and individual qualitative interviews | 297 women |
| Black, 2013 | Health-care providers | 15 countries across Latin America, USA, Europe, Canada and Australia | Online survey | 1862 health care professionals (=average country response rate 18%) |
| Black, Sakhaei and Garland, 2010 | Health-care providers | Australia and New Zealand | Postal survey | 701 OB/GYNs (=response rate 67%) |
| Borrero et al, 2013 | Lay people | USA | Data from the 2009 National Survey of Reproductive and Contraceptive Knowledge | 903 men aged 18-29 (response rate not reported) |
| Bracken and Graham, 2014 | Lay people | UK | Cross-sectional online survey | 502 women aged 18-30 (recruited via social network advertisements) |
| Bratlie et al, 2014 | Health-care providers & lay people | Oslo, Norway | Survey | 359 women aged 16-23 visiting a sexual health clinic (=response rate 90%) and 140 GPs (=response rate 29%) |
| Buhling et al, 2014 | Health-care providers | Eight European countries and Canada | Online survey | 1103 clinicians (OB/GYNs, GPs and FP clinicians including midwives and nurses) (=country response rates to email invites 28% or below) |
| Buhling et al, 2014 | Health-care providers | Germany | Survey | 2016 OB/GYNs (=response rate 21%) |
| Callegari, Parisi and Schwarz, 2013 | Lay people | Pennsylvania, USA | Survey | 1626 women attending a primary care clinic. Women were asked to complete an online or phone survey after their visit (=response rate 19%) |
| Chakraborty et al, 2015 | Health-care providers | Nepal | Survey | 345 randomly selected nurses and auxiliary nurse midwives (non-response not described) |
| De Irala et al, 2011 | Lay people | Germany, France, UK, Romania and Sweden | Household survey | 1137 women (no response rate calculated) |
| Dehlendorf et al, 2011 | Health-care providers | USA | Survey, convenience sampling at professional meetings | 468 physicians |
| Diaz et al, 2011 | Health-care providers | South Carolina, USA | Survey | 133 faculty and residents in South Carolina Area Health Education Consortium (=response rate 53.8%) |
| Ekelund, 2014 | Health-care providers | Sweden | National online survey | 471 midwives and 221 OB/GYNs (=overall response rate of 60%) |
| Fleming, Sokoloff and Raine, 2010 | Lay people | California, USA | Survey | 252 women age 14-27 presenting at an urban family planning clinic (=approximately 70% response rate) |
| Foster et al, 2011 | Lay people | Six cities, USA | Computer-guided survey | 602 women seeking abortion services at six clinics (=61% response rate) |
| Gebremariam and Addissie, 2014 | Lay people | Adigrat, Ethiopia | Cross-sectional household survey | 594 married women sampled through systematic random sampling (=99.5% response rate) |
| Gedeon et al, 2015 | Lay people | Thailand-Burma border | In-person in-depth open-ended interviews | 31 women who obtained IUDs from a clinic |
| Gemzell-Danielsson, 2012 | Health-care providers | Australia, Brazil, Canada, France, Germany, Korea, Mexico, Spain, Sweden and the UK | Anonymous online survey, selected from contact lists of a pharmaceutical company | 1001 health care providers (OB/GYNs, GPs and midwives) (=17% response rate) |
| Gomez and Clark, 2014 | Lay people | USA | Online survey | 382 heterosexual women aged 18-29 (=81% response rate) |
| Gottert et al, 2015 | Lay people | Madagascar | Six small group photonarrative discussions and individual in-depth interviews | Women in the Women's Health Project (WHP) managed by PSI. 18 individuals took part in the group discussions and 12 in the in-depth interviews |
| Gutin et al, 2011 | Health-care providers & lay people | South Africa | Cross-sectional survey | 205 clients (=97% response rate)and 32 health care providers at 12 public sector clinics |
| Habtu et al, 2014 | Lay people | Arnhara Region, Ethiopia | Cross-sectional survey | 986 women using short-acting FP methods in 17 health centres (response rate not reported) |
| Harper et al, 2012 | Health-care providers | USA | National probability survey | 1192 family physicians and OB/GYNs (=response rate 62%) |
| Harper et al, 2013 | Health-care providers | USA | Nationally representative survey | 586 nurse practitioners in primary care and women's health (=69% response rate) |
| Hartman et al, 2012 | Lay people | Northern California, USA | Cross-sectional, random, telephone survey survey | Parent/guardians of 12-17-year old daughters (=35% response rate) |
| Hohmann et al, 2011 | Health-care providers | El Salvador | Anonymous survey | 135 Ministry of Health providers (=95% response rate) |
| Hubacher et al, 2013 | Lay people | Kenya | Survey | 671 women seeking contraception at 6-12 weeks postpartum (=91% response rate) |
| Hubacher et al, 2014 | Health-care providers | Kenya | Pre-post comparison and survey of clinicians | Service statistics from 15 mobile outreach teams (26,070 copper IUD insertions, 40,146 implant, 1030 LNG-IUD), interviews with 27 clinicians |
| Hubacher et al, 2015 | Lay people | Kenya | Cohort study | 313 postpartum women using implant, LNG IUS and copper IUD (response to cohort participation not reported) |
| Jost et al, 2014 | Lay people | France | Postal survey | 5963 representative sample of women aged 15 to 45 (=60% usable response rate) |
| Kavanaugh et al, 2013 | Health-care providers & lay people | USA | Telephone interviews, focus group discussions (FGDs) and in-depth interviews (IDIs) | Telephone interviews with administrative directors at 20 publicly funded facilities, 6 FGDs at 6 of these with facility staff, 48 IDIs with facility clients aged 16-24 |
| Khan and Shaikh, 2013 | Health-care providers & lay people | Rawalpindi, Pakistan | Focus group discussions (FGDs) and in-depth interviews (IDIs) | 6 FGDs with community women and 12 IDIs with FP providers |
| Kohn et al, 2012 | Health-care providers | New York, USA | Self-administered survey | 162 staff at New York City school-based health centers, including clinicians and non-clinicians (=90% response rate) |
| Lessard et al, 2012 | Lay people | Six cities, USA | Survey | 574 women seeking abortions at 6 clinics (=61% response rate) |
| Lete and Perez-Campos, 2014 | Health-care providers & lay people | Spain | Nationwide survey of a representative sample | 2900 women (= exact response rate unclear but less than 50%) and 300 female health care providers (=82% response rate) |
| Luchowski et al, 2014 | Health-care providers | USA | Postal survey | 1221 fellows of the American College of Obstetricians and Gynecologists (= response rate 45.8%) |
| Madden et al, 2010 | Health-care providers | Saint Louis, USA | Postal survey | 184 clinicians providing obstetric and gynecologic care (= 73.7% response rate) |
| Madden et al, 2015 | Lay people | USA | Cross-sectional, self-administered survey | 2590 women enrolled into the Contraceptive CHOICE project, a cohort study (all participants in the cohort completed the survey but representativeness of the cohort not reported) |
| Marshall and Gomez, 2015 | Lay people | USA | The 2009 National Survey of Reproductive and Contraceptive Knowledge | 903 young men (response rate not reported) |
| Marvi and Howard, 2013 | Lay people | Karachi, Pakistan | Qualitative interviews | 20 women, potential contraceptive users living within a health centre catchment area |
| Michie et al, 2013 | Lay people | Scotland, UK | Anonymous, self-administered questionnaire | 106 women requesting an abortion (=85% response rate) |
| Moreau et al, 2013 | Health-care providers & lay people | France | Data from a national population-based survey and a survey of physicians | 3563 women at potential risk of an unintended pregnancy (=response rate 80%) and 364 GPs and 401 OB/GYNs working privately (=42% respose rate) |
| Moreau et al, 2013 | Lay people | France | Data from 3 cross-sectional national probability surveys | 4406 women aged 15-29 (response rate not reported) |
| Morse et al, 2013 | Health-care providers | South Africa and Zimbabwe | Nationally representative surveys | 1444 nurses and physicians (=response rate 73%) |
| Potter, Rubin and Sherman, 2014 | Lay people | New York, USA | Qualitative semistructured interviews | 21 adolescents |
| Rubin et al, 2010 | Lay people | New York, USA | Semistructured interviews | Convenience sample of 40 women |
| Rubin et al, 2011 | Health-care providers | USA | Survey | 3500 family physicians (=25% response rate) |
| Rupley et al, 2014 | Health-care providers | Kumasi, Ghana | Cross-sectional survey, self-administered | 91 providers (specialists, residents, house officers and nurse midwives) (=response rate 65%) |
| Schmidt et al, 2015 | Lay people | St Louis, USA | Focus group discussions (FGDs) and in-depth interviews (IDIs) | Thirteen FGDs and 7 IDIs with 43 young women |
| Spies et al, 2010 | Lay people | A Midwestern state, USA | Telephone survey and focus groups | 543 women aged 18-30 surveyed (=32% telephone response rate and 75% cooperation rate) and 18 focus groups (106 participants) |
| Tang et al, 2013 | Health-care providers | USA | Web-based survey | 699 OB/GYN residents (=response rate 36%) |
| Tumlinson, Okigbo and Speizer, 2015 | Health-care providers | Kenya | Survey | 676 service providers in 273 health care facilities (13 facilities refused participation or had incomplete surveys) |
| Tyler et al, 2012 | Health-care providers | USA | Survey | 635 office-based providers (physicians) and 1,323 Title X clinic providers (physicians, physician assistants, certified nurse midwives, nurse practitioners, and nurses) (=response rates 45% to 76%) |
| Ugaz et al, 2012 | Health-care providers | Bangladesh | Survey | 385 nuses, general practitioners and OB/GYN specialists (=response rate 84%) |
| Weisberg et al, 2013 | Health-care providers & lay people | Australia | Survey | 200 women (recruitment via an online survey panel, response rate not reported) and 162 GPs (=10% response rate) |
| Weston et al, 2012 | Lay people | Chicago, USA | Qualitative interviews | 20 adolescent mothers completed 4-5 interviews |
| White et al, 2013 | Lay people | Texas, USA | Semi-structured interviews | 120 women (criterion-based subsample of a longitudinal study) (1 woman declined to participate) |
| Yinger et al, 2013 | Health-care providers & lay people | Kamdal, Pursat and Battambang provinces, Cambodia | Focus group discussions and key informant interviews | 104 women, 37 men and 31 service providers |
